# Lifestyle advice, processes of care and glycaemic control amongst patients with type 2 diabetes in a South African primary care facility

**DOI:** 10.4102/phcfm.v12i1.2163

**Published:** 2020-03-24

**Authors:** Aswin Kalain, Olufemi B. Omole

**Affiliations:** 1 Division of Family Medicine, Faculty of Health Sciences, University of the Witwatersrand, Johannesburg, South Africa

**Keywords:** lifestyle advice, processes of care, type 2 diabetes, glycaemic control, anthropometric

## Abstract

**Background:**

The influence of processes of diabetes care on glycaemic control is understudied in primary health care (PHC).

**Aim:**

To explore the influence of lifestyle advice, drug regimen and other processes of care on glycaemic control.

**Setting:**

Johan Heyns Community Health Centre, Vanderbijlpark, South Africa.

**Methods:**

In a cross-sectional study involving 200 participants with type-2 diabetes, we collected information on sociodemography, comorbidity, processes of diabetes care, drug regimen and receipt of lifestyle advice. Anthropometric measures and glycosylated haemoglobin (HbA1c) were also determined.

**Results:**

Participants’ mean age was 57.8 years and most were black people (88%), females (63%), overweight or obese (94.5%), had diabetes for < 10 years (67.9%) and hypertension as comorbidity (98%). Most participants received lifestyle advice on one of diet, exercise and weight control (67%) and had their blood pressure (BP) checked (93%) in the preceding 12 months. However, < 2% had any of HbA1c, weight, waist circumference or body mass index checked. Glycaemic control (HbA1c < 7%) was achieved in only 24.5% of participants. Exclusive insulin or oral drug was prescribed in 5% and 62% of participants, respectively. Compared to insulin monotherapy, participants on combined metformin and insulin or metformin, sulphonylurea and insulin were less likely to have glycaemic control. Comorbid congestive cardiac failure (CCF) significantly increased the likelihood of glycaemic control.

**Conclusion:**

There is substantial shortcomings in the implementation of key processes of diabetes care and glycaemic control. Strategies are needed to prompt and compel healthcare providers to implement evidence-based diabetes guidelines during clinic visits in South African PHC.

## Introduction

The prevalence of type-2 diabetes is high worldwide and, in 2015, an estimated 7.0% of those 20–79 years of age in South Africa were affected by the disease, amounting to about 2.3 million people.^1^ Data projections suggest that this number will increase by 140% in the African region by 2040, double the expected global increase by this time.^2^

Type 2 diabetes mellitus (DM) is a devastating condition, responsible for 4 million deaths annually worldwide and 5% of all deaths in sub-Saharan Africa.^3^ Independent of other risk factors, diabetes increases the risk of cardiovascular diseases (CVDs), mostly from macrovascular and microvascular complications.^4^

A cornerstone in the management of type 2 DM is glycaemic control and the processes for achieving this have been well articulated in several guidelines,^1,5^ where clearly defined sets of drug and non-drug processes are recommended. However, not only have several studies reported a pattern of suboptimal glycaemic control, they have also found that healthcare providers across settings often do not optimally comply with recommended processes of care.^1,6,7,8,9^

Sedibeng District Health Services, South Africa, is not an exception to the poor compliance to diabetes care standards. A previous audit in one of its large primary healthcare (PHC) facilities found that different processes of care (including lifestyle advice) were only implemented in between 0% and 49% of patients with type 2 DM.^10^ Also, glycaemic control was achieved in only 30%. These findings have serious implications for primary care in South Africa, considering the global diabetes epidemic and its attendant clinical, public health and socio-economic consequences.

In South Africa, data on glycaemic control, drug treatments, implementation of processes of care and the offer of lifestyle advice by healthcare providers are not integral components of the district health information system. Consequently, this information is unknown. In addition, whilst some previous South African studies^6,7,8,9^ have examined the relationship between a single process of diabetes care and glycaemic control and a few have explored the combined influences of these processes of care on glycaemic control in hospitals, none have done so in PHC facilities. Considering that up to 80% of patients with type 2 DM in South Africa are cared for at the PHC level rather than in hospitals,^10^ further research engagement with the problem of poor quality of diabetes care in this setting is an imperative. This article aims to describe the provision of lifestyle advice, selected processes of care and drug treatment and assess the influence of these factors on glycaemic control in adults with type 2 DM in a large community health centre (CHC), south of Johannesburg. It is envisaged that the study findings could inform interventions for improving the quality of diabetes care and glycaemic control at PHC level of care within South Africa and in similar settings elsewhere.

## Methods

### Design and setting

This was a cross-sectional study conducted at Johan Heyns CHC, Vanderbijlpark, Gauteng province, South Africa. At the time of the study in 2010, this facility served a catchment population of 70 000 and was the referral centre for five PHC clinics. It offered a full range of curative, preventive, promotive and rehabilitation services.

### Sample size and sampling

All patients with type-2 DM who were 18 years and older and attending the CHC for treatment were eligible for the study. To be included in the study, patients needed to have at least two clinic visits for diabetes management in the last 12 months, must have been fluent in any of the four local languages (Sotho, Zulu, Afrikaans and English) and must have given consent. Patients who had emergencies, mental incapacity or pregnancy were excluded from the study.

As a key study objective, glycaemic control was used to determine the sample size. Based on a previous audit in the study setting,^11^ it was assumed that 30% of participants will have glycaemic control. Using an 80% power of detecting a prevalence of glycaemic control within 10% of the hypothesised value and a significance level of 0.05, the required sample size was estimated to be 153. Adjusting for potential loss to follow-up in up to 20% of patients, the final sample size of 191 was rounded up to 200.

Recruitment and sampling were performed as follows: clinic visits for chronic diseases were based on daily booking from Monday to Thursday, excluding public holidays. Medical records of all adult patients with chronic illnesses who had appointments for the next day were retrieved the day before by the medical records clerks. These were inspected by the first author for compliance with all inclusion criteria except consent and language requirements. Each of these patients was consecutively approached for recruitment into the study on the day of their clinic appointment by two research assistants. Whenever a patient was excluded or did not consent, the next consecutive patient was approached. Sampling continued over a period of 6 weeks when the sample size was achieved. Patients who declined continued with usual medical care.

### Measurement tools and data collection

Two measurement tools were used:

- The first tool was a structured questionnaire developed de novo and administered by the two trained assistants. It collected information on sociodemography, duration of diabetes, receipt of advice from a healthcare provider on any of weight control, exercise and diet measures. The first author performed anthropomorphic measurements comprising waist circumference (WC), height, weight and body mass index (BMI) at the time of the interview and recorded on the questionnaire.
- The second tool was a data collection sheet that was used by the researcher to extract information on current drug treatment for diabetes, co-existing medical conditions and the performance of blood pressure (BP), blood glucose, HbA1c, BMI, weight and WC during clinic visits in the last 12 months. Data extraction was carried out at a later convenient time. Glycaemic control was assessed with the HbA1c value not older than 1 month that was available in the records. Where this was not available, blood was drawn for an HbA1c test after the interview.

Each participant’s clinic reference number was recorded on the interview sheet to ensure access to clinical records later and avoid multiple sampling. The reference number was later expunged after the required information had been extracted.

### Data analysis

All analyses were conducted using STATA release 12 (Stata Corp LP, College Station, TX, USA) statistical software. Descriptive statistics was used to summarise variables as means and medians for continuous variables and proportions and frequencies for categorical data. The comparison of means was carried out using analysis of variance (ANOVA) and where this was precluded because of non-homogeneity of variances, a non-parametric test, the Kruskal–Wallis rank test, was applied. Where appropriate, continuous data were recoded as categorical data and analysed accordingly. Associations of glycaemic control were initially explored using Chi-squared test to identify possible outcome predictors amongst explanatory variables. Subsequently, all significant covariates were subjected to univariate logistic regression analysis to derive crude odds ratios for strengths of association. Finally, composite parsimonious explanatory models with adjusted odds ratios for glycaemic control were constructed through multivariate stepwise logistic regression to identify predictors of glycaemic control. A two-tailed *p* < 0.05 was considered statistically significant. The main outcome measures included the following:

- the proportion of participants that achieved glycaemic control, reported receipt of lifestyle advices and had processes of care performed during clinic visits (weight, BMI, WC and BP) – according to the Society for Endocrinology, Metabolism and Diabetes of South Africa (SEMDSA) care standards^11^
- the proportion of participants on different treatment regimens
- sociodemographic, lifestyle, processes of care and treatment variables that are significantly associated with glycaemic control.

### Pilot study

After ethics approval and permission were obtained, a pilot study was undertaken at the research site to refine the quality and structure of the questionnaire, as well as to uncover potential problems regarding sensitivity of questions, ensure uniformity in the administration of questionnaires by the research assistants and assess the feasibility of the study procedures. Ten patients who satisfied the required criteria were chosen for the pilot study. These individuals were excluded from the main study.

### Ethical consideration

Ethics approval was obtained from the Human Research Ethics Committee (Medical) of the University of the Witwatersrand (clearance number: M080635, 15-07-2008). Permission was also obtained from the Sedibeng district management. Data analysis was anonymous as clinic numbers that were recorded on questionnaires to facilitate subsequent linkage to patients’ medical records were later expunged.

## Results

A total of 200 participants were interviewed. Participants’ sociodemographic and comorbidity profiles are presented in Table 1. The mean age was 57.8 years (range: 20–84 years). Most of the participants were blacks, women, aged 50–64 years, had < grade 12 education and derived a form of regular income from employment or government grants.

**TABLE 1:** Participants’ sociodemographic and clinical characteristics.

Categories	Frequency	
	*n*	%
**Demographic parameters†**		
Age group (years) (*n* = 200)		
< 35	3	1.5
35–49	31	15.5
50–64	115	57.5
65–79	49	24.5
80+	2	1.0
Sex (*n* = 194)		
Female	122	62.89
Male	72	37.11
Race (*n* = 200)		
Black people	175	87.5
Indian people	1	0.5
White people	24	12.0
Residence (*n* = 200)		
Catchment area	53	26.5
Outside catchment area	147	74.5
Education (*n* = 199)		
Primary	68	34.2
Secondary	95	47.7
Matric	8	4.0
Tertiary	3	1.5
None	25	12.6
Employment status (*n* = 200)		
Unemployed	81	40.5
Pensioner	59	29.5
Employed	56	28.0
Unspecified	4	2.0
**Duration of Diabetes (years) (*n* = 193)**‡		
Less than 10	131	67.88
10 – 19	54	27.98
20 – 29	7	3.63
More than 30	1	0.51
Presence of comorbid conditions (*n* = 191)		
Present	177	97.7
Absent	14	2.3
Types of comorbid conditions (*n* = 191)§		
Hypertension	176	92.15
CCF	19	9.95
Other	16	8.32

† , Mean (s.d.): 57.8yrs (9.96);

‡ , Mean (s.d.): 7.97yrs (5.56).

§ , Totals may not add up to 200 since some patients had > 1 condition; CCF, Comorbid congestive cardiac failure.

The mean time since diagnosis of diabetes was 7.97 years, with most participants (67.9%) reporting having the disease for < 10 years. Most of the participants (97.7%) had at least one comorbid condition, of which hypertension was the most common (92.2%). Most of the participants (94.5%) were overweight, with 95% and 83% of men and women, respectively, having increased WC.

The proportions of participants on which healthcare providers performed processes of care are shown in Table 2. Except for BP monitoring, which was performed in 93.3% of participants, the implementation of other processes of care was suboptimal. Finger-prick random blood glucose monitoring was performed in 76.7% of participants and 1.6% had the required HbA1c measurements. Approximately 67% of participants received lifestyle advice from a healthcare provider on all of exercise, diet and weight control, with 79% receiving advice on at least one of these lifestyle measures.

**TABLE 2:** Rate of implementation of processes of care and lifestyle advice.

Processes of care	Frequency (*n* = 193)	%
• BP measurement	180	93.26
• Weight	0	0.00
• HbA1c	3	1.55
• BMI	3	1.55
• Waist circumference	0	0.00
**Receipt of lifestyle advice**		
• No advice	42	21.0
• Diet, exercise and weight	127	67.0
• Diet alone	14	7.00
• Diet and weight	8	4.00
• Exercise and diet	8	4.00
• Exercise and weight	1	0.50

BP, blood pressure; BMI, body mass index.

Table 3 shows treatment types and regimens. Most of the participants were prescribed oral hypoglycaemics (62.8%). Of the oral medications, metformin was the most commonly prescribed medication (89.5%) and mostly combined with sulphonylurea.

**TABLE 3:** Diabetes treatment regimens.

Treatment type combinations	Frequency (*n* = 191)	%
• oral only	120	62.83
• oral and insulin	62	32.46
• insulin only	9	4.71
**Medication class combinations (*n* = 191)**		
• metformin + sulphonylurea	93	48.69
• insulin + metformin	30	15.71
• insulin + metformin + sulphonylurea	27	14.14
• metformin only	21	10.99
• insulin only	9	4.71
• sulphonylurea only	6	3.14
• insulin + sulphonylurea	5	2.62

The median HbA1c was 8.4% (range: 4.8% – 14.6%) and glycaemic control (defined as HbA1c < 7%)^12^ was achieved in only 24.5% of participants (Table 4).

**TABLE 4:** Mean HbA1c and glycaemic control.

HbA1c	*n*	%	Median HbA1c	%	*p*
< 7% (Controlled)	47	24.5	6.1	-	-
≥ 7% (Uncontrolled)	145	75.5	9.0	-	0.000

**Totals**	**192**	**100**	**8.4**	**4.8–14.6**	-

On univariate analysis, of all sociodemographic, clinical, lifestyle advice and treatment variables, only race (*p* = 0.01), area of residence (*p* = 0.01) and treatment regimen (*p* = 0.03) were significantly associated with glycaemic control. In the final multivariate regression analysis (Table 5), compared to insulin monotherapy, a combination of insulin and metformin (odds ratio [OR] = 0.22; *p* = 0.02) or metformin, sulphonylurea and insulin (OR = 0.19, *p* = 0.03) conferred lower odds of glycaemic control. Compared to those who did not have it, participants with co-morbid CCF were three times more likely to have glycaemic control (OR = 3.17; *p* = 0.04).

**TABLE 5:** Predictors of glycaemic control.

Variable	Categories	Adjusted OR	*p*
Medication class	Insulin only	1	-
	Insulin and metformin	0.2161132	0.020
	Insulin, metformin and sulphonylurea	0.1858542	0.027
CCF	Absent	1	-
	Present	3.172564	0.035

CCF, Comorbid congestive cardiac failure.

## Discussion

This study found that although most of the participants reported receiving lifestyle advices, healthcare providers’ compliance to standards of key processes of diabetes care and glycaemic control was poor. Whilst these findings reiterate the pervasive suboptimal diabetes care in many clinical settings,^1,13,14^ they have serious clinical, public health and economic implications for South African PHC, including poor clinical outcomes, increased complication rates, increased socio-economic burden, healthcare costs, loss of productivity and reduced quality of life.^15^ To this end, interventions that assist healthcare providers to comply to processes of diabetes care and those that optimise glycaemic control are, therefore, urgent clinical and public health imperatives for South African PHC.

The finding that most of the participants in this study (79%) reported receiving advice on all three components of lifestyle is consistent with studies conducted elsewhere^16^ and demonstrates an improvement compared to at least one other South African study in PHC that reported rates of as low as 32%.^7^ The high rate of offering lifestyle advice in the current study is a welcome finding, considering that the majority of participants were overweight or obese and would benefit from advice in reducing their cardiovascular (CV) risk. Although specialised forms of lifestyle counselling are beneficial for good glycaemic control,^17,18^ no significant relationship was found between receipt of lifestyle advice and glycaemic control in this study, suggesting the need for strategies to assist patients in carrying out the advice at the community level. It should, however, be noted that receipt of advice was based on self-reports and prone to information bias. In addition, we did not assess the nature, content and frequency of lifestyle advice or the category of healthcare providers who delivered the advice.

The finding that 90% of participants had at least one co-morbidity is already confirmed in the literature^19^ and underscores the importance of screening and managing such risks, integrated within diabetes management. This is more so that each co-morbidity tends to be an independent risk for CVD and concurrent risks may exponentially increase the overall risk of CVD in a patient with diabetes.^20^

The pattern of medication use shown in Table 3 has several clinical implications: firstly, it shows a higher use of insulin and a lower use of oral medications compared to other South African studies,^8^ and may suggest lower inertia amongst healthcare providers in this study to initiate insulin. Secondly, the common use of metformin most likely reflects good healthcare providers’ adherence to the South African diabetes guidelines and is thus a welcome practice, especially that metformin is associated with improved lipids profile, little or no risk of hypoglycaemia and reduced macrovascular complications.^1,12^ Thirdly, although it is recommended that sulphonylureas should be continued if basal insulin is initiated but stopped in the case of biphasic insulin,^1,12,21,22^ the long-term use of sulphonylureas may be associated with weight gain, reduced β-cell function and poor metabolic control in type 2 DM. Close monitoring that enables prompt identification of poor glycaemic control is therefore imperative. Lastly, although a small proportion, participants on insulin monotherapy in this study may benefit from reduced polypharmacy, preservation of β-cell function and good glycaemic control. However, insulin monotherapy is recommended only after failure of oral treatment in type 2 DM.^22^

The finding of poor glycaemic control in this study aligns with previous South African studies that have reported glycaemic control in 2.6% – 33.0% of patients with type 2 DM.^6,8^ This pervasive poor glycaemic control signifies that the risk of CVD in South African PHC is substantial, especially that other CV risk factors such as hypertension and hyperlipidaemia are highly prevalent but also sub-optimally managed.^20^ A strong turnaround strategy is, therefore, needed for diabetes care and should include interventions at the individual, health facility and community levels. Interventions at facility level should focus on improving the clinical effectiveness of the PHC team in implementing evidence-based diabetes management guidelines and achieving target treatment outcomes. Small gains in clinical effectiveness have great potential for improving clinical outcomes, considering that a 1% reduction in HbA1c levels results in a significant reduction in microvascular complications.^23,24^

The increased odds of glycaemic control amongst participants with co-morbid CCF have been reported by previous studies^25,26^ and confirm suggestions that healthcare providers tend to aim at tighter control in patients with multiple comorbidity than in those without.^27^ Furthermore, concordant comorbidities often share similar pathophysiological pathways and treating one may result in the benefits of the other.^19,20^

The lower odds of glycaemic control associated with combinations involving insulin as compared to insulin alone in this study contradict the findings in the literature,^28^ which may be because of healthcare provider’s inertia in adding insulin and delayed recognition of treatment failure in patients started on oral hypoglycaemics first. This is supported by the finding that the cohort on insulin combined with oral drugs had a median duration of 7 years since diagnosis and were on insulin for only a median of 16 months, reiterating the fact that insulin was added nearly 6 years after initial diagnosis as opposed to the suggestion that most patients usually remain on oral monotherapy for a median of 4 years before treatment adjustment is made.^29^ Furthermore, the poor monitoring of HbA1c by healthcare providers in the study supports our thought that treatment failure was not identified promptly for timely treatment intensification and hence the resultant poor glycaemic control. Lastly, differences in patients’ and healthcare providers’ treatment priorities possibly result in implicit collusion and unspoken contracts to continue oral agents for as long as possible,^18^ resulting in delayed treatment intensification and persistent poor glycaemic control.

To sum up, the overwhelmingly poor compliance of healthcare providers to most processes of care standards in this study indicts diabetes care in South African PHC and has implications for patients, the healthcare provider and the healthcare system: firstly, healthcare providers’ failure to identify gaps in care prevents prompt interventions that may delay the onset of complications. This, in turn, may adversely affect the patient’s socio-economic productivity and quality of life. Secondly, improvements in adherence to guidelines require multi-faceted quality improvement cycles and lack of monitoring prevents the recruitment of resourceful health team members for the diverse benefit of the patient. Although the adequate implementation of processes of care has attendant implications on resources by way of added costs of laboratory investigations and referrals, implementing simple processes such as HbA1c, BP, BMI and WC monitoring has potential for enormous clinical benefits, requires no special skills or equipment and ought to be routine clinical practice at PHC level. To this end, diabetes clinic visits in South African PHC need to be restructured in such a way that processes of care are considered as vital signs and deviations from standards should prompt healthcare providers to implement corrective measures, including intensifying treatments for optimal glycaemic control.

There are potential limitations of this study. As similarity in characteristics between participants and patients who chose not to participate was not checked, selection bias could have been encountered. The non-documentation of any process of care in patient medical record was considered as non-performance and could have underestimated healthcare providers’ compliance. Lastly, the possibilities of recall bias as well as the possibility of the Hawthorne phenomenon could have resulted in information bias regarding reporting receipt of lifestyle advice. That notwithstanding, this study illuminates the provision of diabetes care in a typical South African PHC facility and the combined influence of lifestyle advice, processes of care and drug treatment on glycaemic control in this setting. In doing so, it provides data based on which interventions for improving the quality of diabetes care may be planned.

In conclusion, there are substantial shortcomings in healthcare providers’ compliance to key processes of diabetes care and glycaemic control in the study setting. Considering the diabetes epidemic and its implications, strategies are direly needed to promote strict adherence by healthcare providers to the processes of diabetes care recommended in evidence-based diabetes guidelines for South African PHC.
